# Epidemiological Features and Outcomes of COVID-19 in Patients With and Without Cardiovascular Disease

**DOI:** 10.34172/aim.2023.48

**Published:** 2023-06-01

**Authors:** Elahe Piraee, Habibollah Azarbakhsh, Ghulamraza mohammadyan, Leila Moftakhar, Aliasghar Valipour

**Affiliations:** ^1^Social Determinants of Health Research Center, Yasuj University of Medical Sciences, Yasuj, Iran; ^2^Ahvaz Jundishapur University of Medical Sciences, Ahvaz, Iran; ^3^Department of Biostatistics and Epidemiology, Faculty of Public Health, Kerman University of Medical Sciences, Kerman, Iran; ^4^Abadan University of Medical Sciences, Abadan, Iran; ^5^Department of Public Health, Abadan University of Medical Sciences, Abadan, Iran

**Keywords:** Cardiovascular disease, COVID-19, Mortality

## Abstract

**Background:**

Cardiovascular diseases (CVDs) are known as an important group of risk factor for progression of the Coronavirus-19 disease (COVID-19). The present study compared epidemiological features and outcomes in COVID-19 patients with CVDs versus those without CVDs.

**Methods:**

This is a retrospective study performed on 1497 patients with CVDs and 26926 patients without CVDs, all of whom were confirmed to have COVID-19. All clinical signs and comorbidities were investigated in the subjects. Mann-Whitney U test and Pearson’s Chi-square test were applied to compare mortality between the groups. Logistic regression was used to identify the predictors of mortality among patients.

**Results:**

The mean age of COVID-19 patients with underlying CVD was 60 years. Totally, about 5.3% of the individuals under study had CVD. Also, 21.6% of all deaths occurred in COVID-19 patients with CVD. Cough, fever, shortness of breath, muscle pain, and underlying diseases such as diabetes, hypertension, chronic liver and kidney disease, chronic lung disease, and immunodeficiency were significantly higher in patients with CVD than those without CVDs. The odds of death in COVID-19 patients were 1.9 times higher with underlying CVD, 2.1 times with diabetes, 3.4 times with hypertension, 1.9 times with immunodeficiency, and 2.3 times with chronic liver and kidney disease.

**Conclusion:**

CVDs are a serious threat to COVID-19 patients because they increase mortality among these patients. As a result, preventive and therapeutic strategies must be developed for these vulnerable groups, who will be prone to higher mortality.

## Introduction

 The coronavirus-19 disease (COVID-19) is a type of severe acute respiratory syndrome, which is caused by a beta-coronavirus (SARS-CoV-2 virus). This disease was first reported in China in December 2019.^1,2^ Severe acute respiratory syndrome coronavirus 2 (SARS-CoV-2) targets the respiratory tract and is clinically similar to severe acute respiratory syndrome of coronavirus (SARS-CoV) and Middle East respiratory syndrome coronavirus (MERS-CoV).^3^ The most common symptoms of this disease are fever, fatigue, and cough, followed by anorexia, myalgia, shortness of breath, etc.^4-8^

 Patients with COVID-19 show significant differences in length of hospitalization, disease progression, and prognosis.^9^ Several meta-analyses have shown that COVID-19 patients with cardiovascular diseases (CVDs), hypertension, diabetes, chronic kidney disease, congestive heart failure, and cancer are at higher risk of death compared to those without these diseases.^1,2,10,11^ Among these diseases, CVDs are the leading cause of death in the world, and account for approximately 17.9 million deaths annually. CVDs are a group of disorders of the heart and blood vessels, including coronary heart disease, rheumatic heart disease, cerebrovascular disease, and others. More than four out of five CVD deaths are due to heart attack and stroke, and one third of these deaths occur prematurely in individuals under 70 years of age.^12^

 In addition, CVDs increase the risk of death among hospitalized COVID-19 patients.^13,14^ In a study on 44,672 COVID-19-confirmed patients in Wuhan, the mortality rate in patients with CVD was reported as 10.5%, while the overall mortality rate was 2.3%.^15^

 CVD is an important risk factor for rapid progression in COVID-19; hence, more acute medical care and services should be provided to COVID-19 patients with CVD. The present study was conducted to investigate and compare the epidemiological feature and outcomes of COVID-19 patients with CVD versus those without CVD.

## Materials and Methods

 In this retrospective observational study, we recruited 28 426 patients with COVID-19 who were admitted to the educational hospitals of Abadan University of Medical Sciences, Southwestern Khuzestan, Iran between February, 2020 and December, 2020. The total population in this region was estimated at 627 970 people, according to the national census in 2020. COVID-19 infection should be confirmed by RT-PCR test on nasal and throat swab samples or definite evidence on high resolution computed tomography (HRCT) imaging. Hospitalized patients were those with severe symptoms such as shortness of breath and chest pain, as well as a saturated oxygen levels < 90%. In addition, some patients who had saturated oxygen levels between 90 and 93% were either admitted to hospital or paced in home quarantine, as judged by the physician according to their clinical condition and underlying diseases. In the region under study, there were only two hospitals, and all stages of hospitalization of patients and testing were performed according to the national guidelines of COVID-19 management, similar to other medical centers in the country. The clinical and demographic information of patients, as well as the outcome of each patient, were recorded.

 After collecting data, duplicate cases were identified and removed based on the national ID number. Patients were divided into two groups: CVD (n = 1497) and non-CVD (n = 26926). First, demographic features, symptoms and underlying conditions were compared between the two study groups; then, the effect of underlying conditions on mortality was examined. Finally, the subset of deceased subjects from the two study group were compared in terms of clinical signs and underlying conditions. The studied variables were age, gender, job, outcome (death vs. survival), symptoms (i.e. shortness of breath, fever, cough, loss of smell and taste, fatigue, muscle pain, gastrointestinal symptoms, sore throat, headache, dizziness, and bloodshot eyes) as well as underlying diseases (i.e. hypertension, diabetes, CVDs, kidney disease, liver disease, chronic lung disease, immunodeficiency, and thyroid disease).

###  Statistical Analyses

 We used median (interquartile range [IQR]) and frequency (percent) to describe the quantitative and qualitative variables, respectively. Group comparison was carried out using Pearson’s chi-square test and Fisher’s exact test for qualitative variables and Mann-Whitney U test for the quantitative variable (age). The normality of the quantitative variable (age) was assessed by Kolmogorov-Smirnov test. We first performed univariate analysis to determine the factors associated with mortality among patients with COVID-19. Then, variables with *P* value < 0.2 were entered into multivariate logistic regression. Data analysis was performed using SPSS version 20.0 and Excel spreadsheet 2016. Statistical significance level was also considered at 0.05

## Results

 During the study period, 28,426 cases of COVID-19 were identified. The median age of patients was 38 [29–50] years, and 56.2% of the subjects were males. Most of the patients (73.2%) mentioned contact with a suspected or definite case of COVID-19. Also, 939 (3.3%) of the patients died, of whom 203 subjects (21.6%) had CVD (Table 1).

**Table 1:** The Characteristics of COVID-19 Patients with or without Cardiovascular Disease

**Variable**	**Total (N=28426) No. (%)**	**Cardiovascular (n=1497) No. (%)**	**Non- Cardiovascular (n=26926) No. (%)**	***P***** Value**
Age, median (IQR)	38 (29-50)	60 (49-70)	37 (29 – 49)	< 0.001
Gender				
Male	15970 (56.2)	751 (50.2)	15219 (56.5)	< 0.001
Female	12456 (43.8)	746 (49.8)	11710 (43.4)	
Symptoms				
Fever	15651 (55.1)	948 (63.3)	14703 (54.6)	< 0.001
Cough	16420 (57.8)	898 (60.0)	15522 (57.6)	< 0.001
Dyspnea	10092 (35.5)	714 (47.7)	9378 (34.8)	< 0.001
Muscular pain	4299 (15.12)	243 (16.2)	4056 (15.1)	< 0.001
Diarrhea	1639 (5.)	90 (6.0)	1549 (5.8)	< 0.001
Runny nose	62 (0.21)	1 (0.1)	61 (0.2)	0.198
Sore throat	685 (2.4)	30 (2.0)	655 (2.4)	0.290
Anorexia	91 (0.3)	3 (0.2)	88 (3)	0.510
Headache/Vertigo	776 (2.7)	42 (2.8)	734 (2.7)	0.805
Fatigue	298 (1.1)	19 (1.3)	279 (1.0)	0.380
Eye redness	16 (0.1)	0 (0.0)	16 (0.1)	0.340
Decreased sense of smell	931 (3.3)	2 (0.1)	929 (3.5)	< 0.001
Decreased sense of taste	863 (3.0)	49 (3.3)	813 (3.0)	0.570
Shiver	1647 (5.8)	61 (4.1)	1586 (5.9)	0.003
Comorbidities				
Hypertension	1579 (5.5)	379 (25.3)	1200 (4.5)	< 0.001
diabetes	2288 (8.0)	520 (34.7)	1768 (6.6)	< 0.001
Immunodeficiency	569 (2.0)	88 (5.9)	481 (1.8)	< 0.001
Chronic kidney, liver disease	425 (1.5)	51 (3.4)	374 (1.4)	< 0.001
Chronic pulmonary disease	746 (2.6)	141 (9.4)	605 (2.2)	< 0.001
History of ICU admission	94 (0.3)	19 (1.3)	75 (0.3)	< 0.001
Travel history	60 (0.2)	0 (0)	60 (0.2)	< 0.001
Exposure to disease	20813 (73.2)	1010 (67.5)	19803 (73.5)	< 0.001
Mortality	939 (3.3)	203 (13.6)	736 (2.7)	< 0.001

 The most common symptoms were cough (57.8%), fever (55.1%), and shortness of breath (35.5%), muscle pain (15.1%), and gastrointestinal symptoms (5.8%). The most common underlying diseases were diabetes (8%) hypertension (5.6%), CVDs (5.3%), immunodeficiency diseases (including cancers) (2%), chronic lung disease (2.6%), and chronic kidney and liver diseases (1.70%) (Table 1).

 In all study participants, 1497 (5.3%) had CVD and 26926 (94.7%) had no CVDs. Compared to non-CVD patients, CVD patients were older (37 [29–49] vs. 60 [49–70] years).

 In addition, other underlying diseases were more prevalent in CVD patients than those without CVD: diabetes (520 [34.7%] vs. 1768 [6.6%]), hypertension (379 [25.3%] vs. 1200 [4.5%]), chronic lung disease (141 [9.4%] vs. 605 [2.2%]), chronic liver and kidney diseases (51 [3.4%] vs. 374 [1.4%]), and thyroid disease (17 [1.1%] vs. 158 [0.6%]) (Table 1).

 A total of 27,277 patients (95.9%) survived COVID-19. Compared to these patients, deceased patients were significantly older (66 [ 55–76] vs. 37 [29–49] years, *P* < 0.001), and had a higher prevalence of chronic diseases including diabetes (*P* < 0.001), hypertension (*P*< 0.001), CKD (*P* < 0.001), chronic liver diseases (*P* < 0.001), chronic pulmonary diseases (*P* < 0.001), immunodeficiency (*P* < 0.001), and thyroid disease (*P* < 0.001) (Table 2).

**Table 2:** Baseline Characteristics of Survivors and Non-survivors Infected with COVID-19

**Variable**	**Total (N=28216)** **No. (%)**	**Survivors (n=27277)** **No. (%)**	**Non-survivors (n=939)** **No. (%)**	***P***** value**
Age, median (IQR)	38 (29-50)	37 (29-49)	66 (55-76)	< 0.001
Gender				
Male	15970 (56.2)	15456 (56.7)	514 (54.7)	0.510
Female	12456 (43.8)	12031 (43.3)	425 (45.3)	
JOB				
Worker	1160 (6.2)	1148 (4.2)	2 (0.7)	< 0.001
Health worker	1974 (10.4)	1958 (7.2)	1 (0.4)	
Employee	3706 (19.5)	3675 (13.5)	5 (1.9)	
Freelance worker	3772 (19.8)	3724 (13.6)	21 (7.8)	
Student, college student	1355 (7.1)	1331 (4.9)	8 (3)	
Disabled, elderly	666 (3.5)	616 (2.3)	48 (17.9)	
Housekeeper	4835 (25.4)	104 (.4)	4686 (25.2)	
Child-neonatal	225 (1.2)	222 (1.2)	1 (0.4)	
Retired	499 (2.6)	32 (11.9)	464 (2.5)	
Unemployed	704 (3.7)	45 (16.8)	648 (3.5)	
Farmer	131 (0.7)	1 (0.4)	130 (0/7)	
Comorbidities				
Diabetes mellitus	2288 (8.0)	2011 (7.4)	277 (29.5)	< 0.001
Hypertension	1579 (5.5)	1326 (4.9)	253 (26.9)	< 0.001
Thyroid disease	175 (0.6)	160 (0.6)	15 (1.6)	< 0.001
Immunodeficiency	569 (2.0)	486 (1.8)	83 (8.8)	< 0.001
Chronic kidney, liver disease	425 (1.5)	379 (1.4)	46 (4.9)	< 0.001
Chronic pulmonary disease	746 (2.6)	682 (2.5)	64 (6.8)	< 0.001

 Moreover, in the CVD group, deceased patients were significantly older than the survivors (69 [61–78] vs. 59 [47–68] years, *P* < 0.001). Risk of fever (*P* = 0.020), dyspnea (*P* < 0.001), muscle pain (*P* = 0.048), hypertension (*P* < 0.001), and diabetes (*P* = 0.010) was higher in deceased patients. Gender, other underlying diseases, and frequency of symptoms showed no significant difference between the two subsets of COVID-19 patients who had CVDs (Table 3).

**Table 3:** Baseline Characteristics of Survivors and Non-survivors in COVID-19 Patients With Cardiovascular Disease

**Variable**	**Total (N=1495)** **No. (%)**	**Survivors (n=1282)** **No. (%)**	**Non-survivors (n=203)** **No. (%)**	***P***** value**
Age, median (IQR)	60 (49-70)	59 (47-68)	69 (61-78)	< 0.001
Sex				
Male	751 (50.2)	648 (50.1)	103 (50.7)	0.830
Female	746 (49.8)	646 (49.9)	100 (49.3)	
Symptoms				
Fever	948 (63.3)	800 (61.8)	148 (72.9)	0.020
Cough	898 (60.0)	761 (58.8)	137 (67.5)	0.180
Dyspnea	714 (47.7)	594 (45.9)	120 (59.1)	< 0.001
Muscular pain	243 (16.2)	224 (17.3)	19 (9.3)	0.048
Gastrointestinal symptoms	90 (6.0)	85 (6.5)	5 (2.5)	0.097
Sore throat	30 (2.0)	24 (1.8)	6 (2.9)	< 0.001
Comorbidities				
Hypertension	379 (25.3)	298 (23.0)	81 (39.9)	< 0.001
Diabetes	520 (34.7)	431 (33.3)	89 (43.8)	0.010
Immunodeficiency	88 (5.9)	75 (5.8)	13 (6.4)	0.870
Chronic pulmonary disease	141 (9.4)	113 (8.7)	28 (13.8)	0.070
Chronic liver, kidney disease	51 (3.4)	40 (3.1)	11 (5.4)	0.190
Exposure to disease	1010 (67.5)	889 (68.7)	121 (59.6)	0.002

 The result of multivariate logistic regression showed that the odds of death in COVID-19 patients were 1.9 times higher with underlying CVD, 2.1 times with diabetes, 3.4 times with hypertension, 1.9 times with immunodeficiency, and 2.3 times with chronic liver and kidney disease compared to patients without these diseases. In addition, with each year increase in age, the odds of mortality rose by 1.003 times (Table 4).

**Table 4:** Predictors of Mortality in Patients With COVID-19 Based on the Results of Multiple Logistic Regression.

**Variable**	**OR (95% CI)**	***P***** Value**
Age	1.003 (1.001-1.004)	< 0.001
Cardiovascular disease	1.952 (1.339-2.846)	0.001
Diabetes	2.194 (1.557-3.092)	< 0.001
Hypertension	3.453 (2.461-4.846)	< 0.001
Immunodeficiency	1.927 (1.088-3.413)	0.025
Chronic kidney/liver disease	2.307 (1.203-4.425)	0.012

OR, odds ratio.

## Discussion

 The present study was conducted on 28,426 COVID-19 patients in southwestern Iran, to compare clinical signs and mortality rate amongst patients with and without CVDs. Even though COVID-19 affects all healthy individuals at any age, there is some evidence that some people with underlying diseases, including CVDs, diabetes, hypertension, etc. as well as older people are more likely to develop a more severe medical condition. Many studies have reported that COVID-19 patients with CVD tend to be more infected with higher rates of ICU admission and mortality.^6,7,10,15-20^

 In this study, 5.3% of COVID-19 patients had CVD, which is inconsistent with the results of similar studies. As such, a meta-analysis on 1,576 people, showed a prevalence of heart disease in patients with COVID-19 as high as 8.4%.^21^ In a meta-analysis by Rodriguez and colleagues, this rate was 14.4%.^22^ In other similar studies, the rate of CVD in COVID-19 patients was reported at 12%,^23^ 16.4%,^24^ 15%,^6^ 27%^25^ and 30%.^26^ The discrepancy among these reports can be due to differences in sample sizes across studies as well as differences in the cultural, economic and health status of the affected areas, which result in different prevalence rates.

 In our study, the rate of ICU admission was higher in patients with CVD than those without CVD (1.3% vs. 0.3%). Many other studies have shown that CVD patients with COVID-19 are more likely to have severe medical conditions and more frequently require ICU admission. Accordingly, the rate of ICU admission of COVID-19 patients with CVD was 25% in China,^7^ 21% in Italy,^27^ and 14% in New York.^28^ In our study, although only 5.3% of COVID-19 patients had CVD, about one-fifth (21.6%) of mortalities occurred in this group of patients. A study in China also found that although 4.2% of COVID-19 patients had CVD, a higher proportion of mortality (18.3%) was attributed to these patients.^29^

 The results of the present study showed that the oddsof mortality in COVID-19 patients with CVD was about twice as high as patients without CVD. Li et al conducted a meta-analysis and reported that the risk of mortality in COVID-19 patients with CVD was 4.85 times higher than those without it. Also, it was estimated at 21.4 times higher in a Chinese study,^17^ and even this figure was doubled in a study on 115 patients in Hong Kong.^18^ Also, the mortality rate of COVID-19 patients with CVD was 10.5% in China.^15^ However, the reasons for the higher mortality rate among CVD patients are still unclear. Nevertheless, some studies have suggested that in the context of COVID-19, CVD is associated with reduced cardiac function and accelerated myocardial infarction. Also, metabolic demand and myocardial demand are increased, resulting in increased mortality in these patients.^30^ Also, CVD patients do not have a stable hemodynamic status, hence left/right ventricular dysfunction and cardiogenic shock would be more likely in COVID-19 infection.^20^ Moreover, inflammatory responses caused by COVID-19 can promote coronary heart disease to acute coronary syndrome, followed by blood clotting and rupture of arteries.^20^ It should be noted that 48.3% of deaths related to COVID-19 have occurred in patients who had both CVD and diabetes, and 39.9% among COVID-19 patients with CVD and hypertension. Also, diabetes and hypertension alone increased the oddsof death by 2.1 and 3.4 times, respectively. On the other hand, it is also known that both diabetes and hypertension are possible risk factors for CVDs, so these can exacerbate heart disease. Further research is warranted to determine the role of each of these diseases in death amongst patients with COVID-19.

 The median age of COVID-19 patients with CVD was higher than COVID-19 patients without CVD (66 years vs. 37 years), which was in accordance with the results of Xie and colleagues who reported a median age of 66 years in COVID-19 patients with CVD and 58 years in non-CVD patients.^31^ This may be due to the fact that older people usually have a weakened cellular and humoral immune system, which leads to lower antiviral response, and subsequently virus replication in the body and increased risk of heart attack.^32^ Also, the risk of hypertension and diabetes, which are risk factors for CVD, increases with age,^33^ and raises the risk of death in these people.

 In our study, the incidence of COVID-19 was almost equal in men and women, while the results of chi-square test showed a significant difference. In another study, it was reported that men were more likely to develop this disease and manifest severe complications.^34^ The results of our study showed that a number of symptoms, such as cough, fever, dyspnea, muscle pain, diarrhea, and sore throat were higher in COVID-19 patients with CVD than those without CVD, which might reflect a more severe form of COVID-19 in patients with CVD.

 Although in our study, laboratory and radiologic results of patients were not available, it must be acknowledged that our study was one of the first studies in Iran examining the clinical characteristics of COVID-19 patients with CVD. Our study was conducted on a large sample size, examined several underlying diseases, and included both mild and severe patients, while many in-line studies were limited to patients with the severe form of COVID-19. Our study also identified the role of underlying diseases and the importance of CVD in the death of patients with COVID-19, and thus, it can help to develop hypotheses for future studies and formulate the necessary strategies in this field.

 In conclusion, according to the findings of this present study, CVDs are a serious threat to COVID-19 patients because they can increase the mortality rate. However, there are still many unknown issues about COVID-19; for example, the exact link between the disease and CVDs is not well understood. Management of COVID-19 poses a huge challenge. As a result, preventive and therapeutic strategies should be developed for high risk groups, especially those with CVDs. It is also suggested that CVD patients must take preventive measures, such as quarantine in home and social distancing, avoiding unnecessary visits to medical centers, and paying attention to the preventive guidelines developed by the experts of the World Health Organization.
